# Impact of SARS-CoV-2 on Aerobic and Anaerobic Capacity in Professional Ice Hockey Players

**DOI:** 10.3390/jcm14103478

**Published:** 2025-05-16

**Authors:** Robert Roczniok, Artur Terbalyan, Przemysław Pietraszewski, Grzegorz Mikrut, Hanna Zielonka, Petr Stastny, Andrzej Swinarew, Daria Manilewska, Kajetan Ornowski, Tomasz Jabłoński, Patrycja Lipińska

**Affiliations:** 1 Institute of Sport Science, The Jerzy Kukuczka Academy of Physical Education, Mikołowska 72A, 40-065 Katowice, Poland; a.terbalyan@awf.katowice.pl (A.T.); g.mikrut@awf.katowice.pl (G.M.); andrzej.swinarew@us.edu.pl (A.S.); dmanilewska@gmail.com (D.M.); ornowskikajtek@gmail.com (K.O.); 2 Faculty of Biomedical Engineering, Silesian University of Technology, Roosevelta 40, 41-800 Zabrze, Poland; hz312613@student.polsl.pl; 3 Department of Sports Games, Faculty of Physical Education and Sport, Charles University, 162 52 Prague, Czech Republic; stastny@ftvs.cuni.cz; 4 Faculty of Science and Technology, University of Silesia, 75 Pułku Piechoty 1A, 41-500 Chorzów, Poland; 5 Faculty of Health Science and Physical Education, Kazimierz Wielki University in Bydgoszcz, 85-867 Bydgoszcz, Poland; tomas82@ukw.edu.pl (T.J.); patlip@ukw.edu.pl (P.L.)

**Keywords:** COVID-19, aerobic capacity, anaerobic capacity, ice hockey

## Abstract

**Background/Objectives:** COVID-19 poses significant physiological challenges for athletes, particularly those engaged in high-intensity intermittent sports such as ice hockey. This study aimed to evaluate the impact of SARS-CoV-2 infection—especially symptomatic cases—on aerobic and anaerobic performance in professional ice hockey players. **Methods:** Fifty athletes from the Polish Hockey League were assigned to three groups: control (CG, n = 13), asymptomatic COVID-19 (NSG, n = 28), and symptomatic COVID-19 with post-infection SpO_2_ < 90% (WSG, n = 9). Each underwent assessments at three time points—pre-preparatory period 2020/2021, post-preparatory period 2020/2021, and pre-preparatory period 2021/2022. Aerobic capacity was measured via a progressive cycle ergometer test (VO_2_max, VO_2_ at lactate threshold [VO_2Lt_], minute ventilation [V’E], breathing frequency [BF], and lactate clearance rate [ΔLa]), and anaerobic capacity via a 30 s Wingate test (relative mean power). **Results:** Compared with CG and NSG, symptomatic athletes exhibited significant post-infection declines in VO_2_max (48.2 ± 2.9 vs. 56.2 ± 6.2 and 54.6 ± 3.9 mL/kg/min; *p* = 0.006, d = 1.56 vs. CG; *p* < 0.024, d = 1.79 vs. NSG) and VO_2Lt_ (*p* < 0.05). Relative mean power also decreased in WSG (*p* < 0.05). In contrast, CG and NSG improved or maintained these metrics over the same period. Symptomatic players showed elevated BF post infection (*p* = 0.022, d = 1.72) and reduced V’E (*p* = 0.035; d = 0.83), while ΔLa was markedly lower (*p* = 0.0004; d = 2.86). **Conclusions:** SARS-CoV-2 infection, particularly symptomatic cases, can significantly impair both aerobic and anaerobic capacity in elite hockey players. Targeted recovery protocols are essential for restoring performance in affected athletes.

## 1. Introduction

Ice hockey is a high-intensity intermittent sport that places significant demands on both the aerobic and anaerobic energy systems. VO_2_max values among elite players typically range from 50 to 60 mL/kg/min, and in NHL prospects, they may reach even 62 mL/kg/min [1,2,3,4,5,6,7,8,9,10]. During competition, approximately 69% of the energy demand is met by anaerobic metabolism, with oxygen debt reaching up to 9.6 L and lactate concentrations ranging between 8–14 mmol/L [2,5,9,11]. Efficient aerobic recovery between shifts is essential for maintaining high performance. The speed of the recovery process determines the speed of utilization of glycolysis byproducts, and thus a decrease in the VO_2_max value below 50 mL/kg/min may limit the player’s ability to regenerate quickly, and in the long run, it limits the ability to maintain high-intensity work during the match and tactical assumptions outlined by the coach. Anaerobic capacity is of key importance to players in ice hockey, which has also experienced a very large increase in performance in recent years, confirming that ice hockey has become a faster game over the years and requires players to work at ever higher intensity [5,7,9,12]. According to Montgomery [4,13], the average playing intensity of a ice hockey player is around 70–90% of HRmax. That is why COVID-19, with its mechanisms, has become a very serious problem for professional players, especially those in whom SARS-CoV2 was symptomatic, including a decrease in SpO_2_ saturation < 90%. In early 2020, the SARS-CoV-2 virus rapidly spread worldwide, leading to disruptions in sports seasons and training schedules across all levels of competition. Beyond the organizational impact, the virus also presented direct physiological challenges for athletes. COVID-19 predominantly affects the respiratory system, leading to impaired pulmonary function, inflammation, and reduced oxygen saturation (SpO_2_), especially in symptomatic individuals. For athletes, this can translate into diminished aerobic performance, slower recovery rates, and limitations in high-intensity capacity.

Studies have shown that even in mild or asymptomatic cases, SARS-CoV-2 can result in subclinical changes in cardiopulmonary function, fatigue, or altered lactate kinetics. In more severe symptomatic cases—especially those with reduced blood oxygen saturation below 90%—the effects may include decreased VO_2_max, reduced ventilatory efficiency, and impaired mitochondrial function. These factors are especially critical in high-intensity intermittent sports like ice hockey, where aerobic capacity and lactate clearance determine recovery between efforts and sustained performance during matches [14,15,16,17,18,19].

Given these potential consequences, monitoring and understanding the effects of SARS-CoV-2 infection on elite athletes is essential for guiding return-to-play decisions and adapting training protocols during and after recovery [20,21].

The fact that the amount of virus particles may vary in different parts of the respiratory system may be important in detecting infection, indicating that breath analysis may be more sensitive than traditional swabs [22]. The aim of this study was to assess the impact of SARS-CoV-2 infection on aerobic (VO_2_max, VO_2Lt_) and anaerobic (Wingate power, lactate clearance) performance in professional hockey players. The analysis compared a control group, an asymptomatic COVID-19 group, and a symptomatic group with SpO_2_ < 90%, at three time points: before and after the 2020/2021 preparatory period, and before the 2021/2022 season.

## 2. Materials and Methods

### 2.1. Participants

A total of 50 ice hockey players from clubs playing in the top league in Poland in the 2020/2021 and 2021/2022 seasons participated in the study. All athletes had a minimum of 12 years of continuous training experience. The average age was 27 ± 6 years. Exclusion criteria were chronic cardiopulmonary disease, acute injury precluding ergometer/Wingate tests, performance-modulating drug use within six weeks, missing ≥1 session, consent withdrawal, or violation of pre-test standardization. The first tests were carried out before the preparatory period in May and June 2020, in the season in which, after frostbite of restrictions, the number of infections with the SARS-CoV-2 pathogen among players increased very much. After performance tests and the start of the preparation period in August, a very large number of players tested positive for COVID-19, which resulted in their quarantine in which practically entire ice hockey teams landed. In the study, the group of players was divided into ice hockey players who were not diagnosed with COVID-19 (CG—control group N = 13), hockey players who were diagnosed with COVID-19 but the disease was asymptomatic (NSG—no symptoms group N = 28), and hockey players with symptomatic disease (WSG—with symptoms group N = 9). Symptomatic cases were defined by self-reported symptoms and confirmed peripheral oxygen saturation (SpO_2_) < 90%, a clinically relevant threshold for hypoxemia. This marker is commonly used to indicate compromised respiratory function and was chosen to differentiate between physiological impact severity. After the first wave of COVID-19 passed, the players continued the preparation period for the season. The second series of performance tests were carried out before the league season (end of September), whose first round, due to the coronavirus, did not start until November 9. The 3rd round of tests was carried out before the preparatory period for the next season in 2021/2022. The tested athletes were informed about the purpose and methods of the research and about the possibility of withdrawing from the research at any stage. Prior to testing, each tested participant underwent a medical examination to rule out the presence of a cold, fever, or any condition that could serve as an exclusion criterion for the study. The research was conducted with the consent of the Ethics Committee for Scientific Research at the Jerzy Kukuczka Academy of Physical Education in Katowice (2/2019) of 14 November 2019. All procedures were carried out according to the Declaration of Helsinki (Table 1).

### 2.2. Research Design

In each of the research periods (before the preparatory period, after the preparatory period, and before the play-off) the same test procedure was used. The study was conducted in a certified functional testing laboratory of the Institute of Sport at the Academy of Physical Education in Katowice, under standard conditions with controlled temperature and humidity. On the first day of the study, body weight and body composition were measured in three study periods using the InBody 220 (Biospace Co., Tokyo, Japan). The measurements were made by a person trained for this purpose, holding a certificate issued by the exclusive distributor of the InBody body composition analyzer in Poland. Body weight was measured in the morning between 9.00 and 10.00, 2 h after breakfast, after using the toilet. Before the measurement, people did not perform any exercises and did not take any medications. The tests were carried out at a temperature of 21 °C. Then, the diagnosis of anaerobic capacity was performed. After 48 h of rest, the diagnosis of aerobic capacity was performed. In the study, a progressive exercise test on a cycle ergometer (Excalibure Sport, Lode, Groningen, The Netherlands) was used, during which VO_2_max was determined, and VO_2_max was determined at the anaerobic threshold. The stress test started with a load of 40 W and included an increase in the load by another 40 W every 3 min. The test continued until the subject declined or was unable to maintain the minimum cadence of 60 rpm. During the test, a cadence of 60–90 rpm was recommended. If the test was terminated before the end of the set load, the maximum load was calculated using the formula proposed by Kuipers et al. [23]. At rest (3 min before the test) and during the test, heart rate (HR), minute ventilation (V’E), respiratory rate (BF), oxygen uptake (VO_2_), and exhaled carbon dioxide (VCO_2_) were continuously recorded using a MetaLyzer 3B-2R (Cortex, Isernhagen, Germany) high-speed gas analyzer. All variables were analyzed during each breath (breath-by-breath method) and presented in averaged 15 s time intervals. The following criterion was used in the assessment of VO_2_max: When the respiratory rate (RER) value was >1.1, at the end of each load (last 15 s), fingertip capillary blood was drawn to determine lactate concentration. The obtained data were used to analyze the kinetics of the concentration of this metabolite in the blood. The lactate concentration was also monitored at 3, 6, 9, and 12 min of recovery. In order to analyze the rate of recovery, ΔLa [mmol/L] was calculated, i.e., the difference in lactate concentration between the 3rd and 12th minute of recovery. Blood lactate determinations were performed using the Biosen C-line Clinic analyzer (EKF-diagnostic GmbH, Barleben, Germany). On the second day, players underwent a standardized protocol for anaerobic testing. After resting lactate measurement, and a 10 min warm-up on a cycle ergometer (Excalibure Sport, Lod, Groningen, The Netherlands), each player completed a 30 s Wingate test with a resistance of 0.85 W/kg. During the test, peak, mean, and minimum power outputs were recorded and normalized to body weight. Capillary blood samples were collected before the test, and at 4 and 8 min post exercise, to assess blood lactate concentration and recovery kinetics. Lactate was analyzed using the Biosen C-line Clinic analyzer (EKF-diagnostic GmbH, Barleben, Germany). The timing of tests was aligned with key phases of the training cycle to assess performance before and after preseason preparation. To reduce the impact of confounding variables, all participants underwent standard medical screening prior to testing. While factors such as psychological stress, nutrition, or training volume were not strictly controlled, players followed club-level programs under similar conditions. Absences from training due to quarantine or illness were recorded but not statistically modeled due to group size limitations.

### 2.3. Statistical Analyses

To characterize the structure of the analyzed variables, basic descriptive statistics were computed, including the arithmetic mean, standard deviation, and 95% confidence intervals for the mean. A repeated measures analysis of variance (ANOVA) was used to determine the significance of differences between groups. Assumptions for ANOVA were verified using the Shapiro–Wilk test (normality), Levene’s test (homogeneity of variance), and Mauchly’s test (sphericity). Where assumptions were violated, corrections were applied (e.g., Greenhouse–Geisser). In cases where significant effects were identified in the ANOVA, post-hoc tests with Bonferroni adjustment were used. Effect sizes (partial η^2^) are reported consistently in both text and tables. The interpretation of η^2^ followed the following criteria: weak effect: (η^2^ = 0.01–0.059); moderate effect: (η^2^ = 0.06–0.137); large effect: (η^2^ > 0.137). Cohen’s d was calculated for all pairwise comparisons and interpreted using conventional thresholds (small = 0.20, medium = 0.50, large = 0.80). An a priori power analysis was performed using G*Power 3.1 software to estimate the minimal required sample size. Assumptions included a medium effect size (f = 0.25), alpha level (α) set at 0.05, and desired statistical power at 0.95. Additionally, a correlation of r = 0.60 among repeated measures and no sphericity correction (ε = 1) were assumed. The analysis indicated a minimal total required sample size of 42 participants. The actual calculated statistical power for the conducted study was 0.963. For all statistical analyses, the significance level was set at α = 0.05. Statistical calculations were performed using the Statistica 13.1 software package (TIBCO Software Inc., Palo Alto, CA, USA).

## 3. Results

The results for the analyzed variables are presented in Table 2.

**Figure 1:**
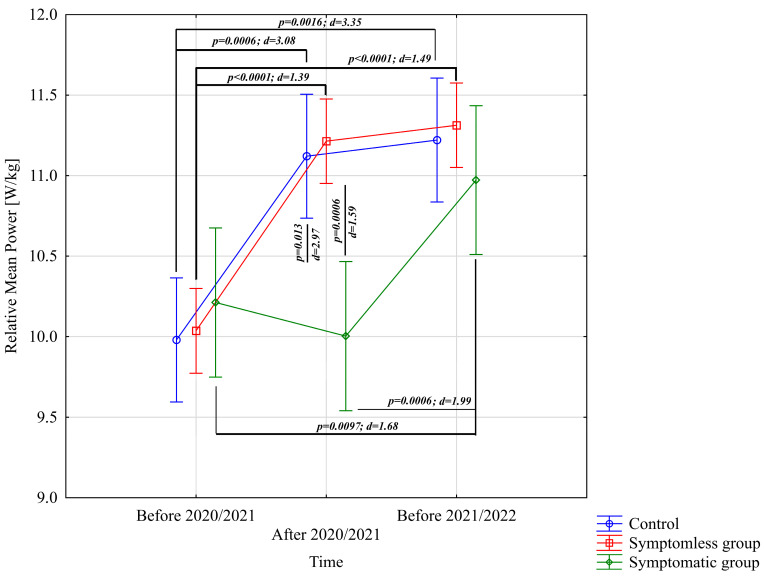
Comparison of mean values and confidence intervals for the Relative Mean Power variable by group and study time. *p*—*p*-value; d—Cohen’s d effect size.

The analysis of the results presented in Table 2 did not provide sufficient evidence to conclude significant differences in time to peak power [s], neither for main effects nor for interactions (*p* > 0.05). In the case of relative peak power, a significant difference was observed for the study time main effect only (*p* < 0.0001; η^2^ = 0.20). The analysis of Relative Mean Power [W/kg] from the Wingate test indicated significant differences for the main effect of study time (*p* < 0.0001; η^2^ = 0.43) and the interaction of group × study time (*p* = 0.00016; η^2^ = 0.21). Post-hoc multiple comparison tests using Bonferroni corrections revealed significantly higher Relative Mean Power [W/kg] results after the preparatory period 2020/2021 and before the preparatory period 2021/2022 in both NSG and CG groups, compared to the results before the preparatory period 2020/2021 (Figure 1). In the WSG SpO_2_ < 90% group, no significant differences were observed between the results before and after the 2020/2021 preparatory period (*p* = 0.99). However, significant differences (increases in Relative Mean Power) were identified between results obtained before the preparatory period 2020/2021 and those before the 2021/2022 period, as well as between post-preparatory 2020/2021 and pre-preparatory 2021/2022 periods (*p* = 0.0006). Significant intergroup differences in Relative Mean Power were noted only after the preparatory period 2020/2021, with the NSG and CG groups achieving statistically significantly higher results compared to the WSG SpO_2_ < 90% group (Figure 1).

**Figure 2:**
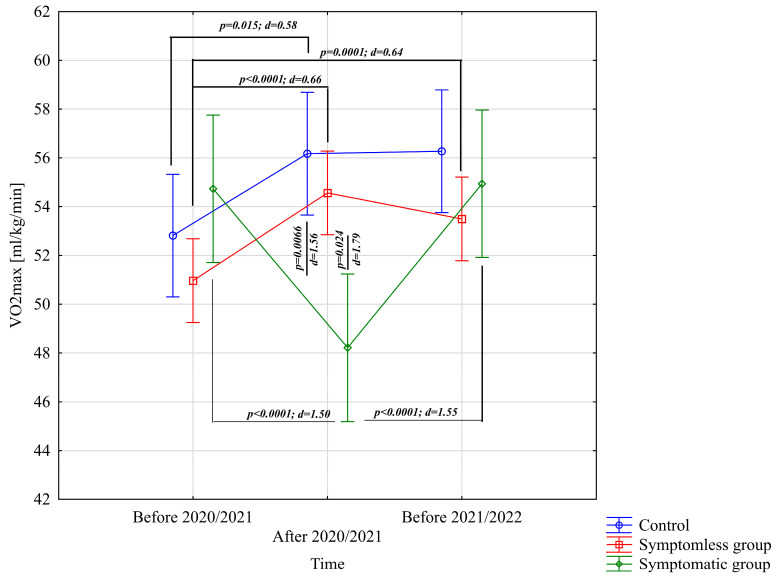
Comparison of means and confidence intervals for the VO_2_max variable by group and study time. *p*—*p*-value; d—Cohen’s d effect size.

The analysis of VO_2_max [mL/kg/min] indicated significant differences for the main effect of study time (*p* = 0.0012; η^2^ = 0.13) and the interaction of group × study time (*p* < 0.0001; η^2^ = 0.43). Analysis of the group × study time interaction for VO_2_max [mL/kg/min] revealed significantly higher results in the NSG group after the preparatory period 2020/2021 and before the preparatory period 2021/2022 compared to the results before the preparatory period 2020/2021 (Figure 2). The CG group demonstrated significantly higher results after the preparatory period 2020/2021 compared to before the preparatory period 2020/2021 (Figure 2). Conversely, significantly lower results were observed after the preparatory period 2020/2021 compared to periods before the preparatory period 2020/2021 and before the preparatory period 2021/2022. Significant intergroup differences in VO_2_max [mL/kg/min] were noted only after the preparatory period 2020/2021, with results in the NSG and CG groups significantly higher compared to the WSG SpO_2_ < 90% group (Figure 2).

**Figure 3:**
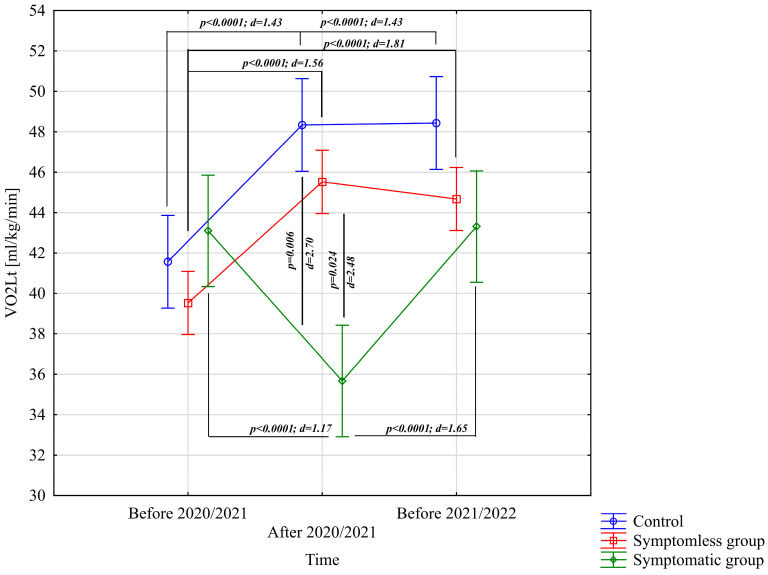
Comparison of mean values and confidence intervals for the VO_2Lt_ variable by group and study time. *p*—*p*-value; d—Cohen’s d effect size.

The analysis of VO_2Lt_ [mL/kg/min] revealed significant differences for the main effect of study time (*p* < 0.0001; η^2^ = 0.46) and the interaction between group and study time (*p* < 0.0001; η^2^ = 0.69). Further analysis of the group × study time interaction for the VO_2Lt_ [mL/kg/min] variable indicated significantly higher values in the NSG and CG groups after the preparatory period 2020/2021 and before the preparatory period 2021/2022 compared to the period before the preparatory period 2020/2021 (Figure 3). After the preparatory period 2020/2021, significantly lower VO_2Lt_ [mL/kg/min] values were observed compared to those recorded before the preparatory periods of both 2020/2021 and 2021/2022 (Figure 3). Significant intergroup differences in VO_2Lt_ [mL/kg/min] were only noted following the 2020/2021 preparatory period, during which the NSG and CG groups achieved significantly higher results compared to the WSG SpO_2_ < 90% group (Figure 3).

**Figure 4:**
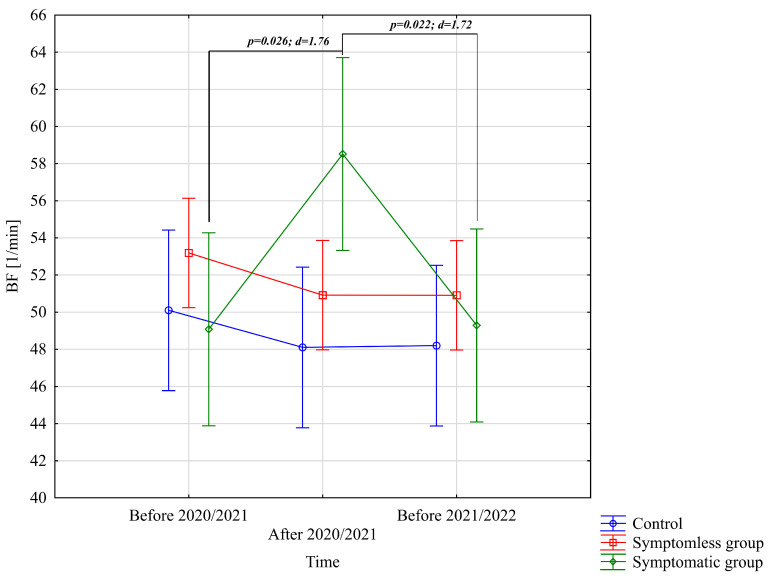
Comparison of mean values and confidence intervals for the BF [1/min] variable by group and study time. *p*—*p*-value; d—Cohen’s d effect size.

The analysis of BF [1/min] revealed significant differences only for the interaction between group and study time (*p* = 0.011; η^2^ = 0.13). Further analysis of the group × study time interaction for the BF [1/min] variable indicated significantly higher values in the WSG SpO_2_ < 90% group after the preparatory period 2020/2021 compared to the periods before the preparatory periods of both 2020/2021 and 2021/2022 (Figure 4).

**Figure 5:**
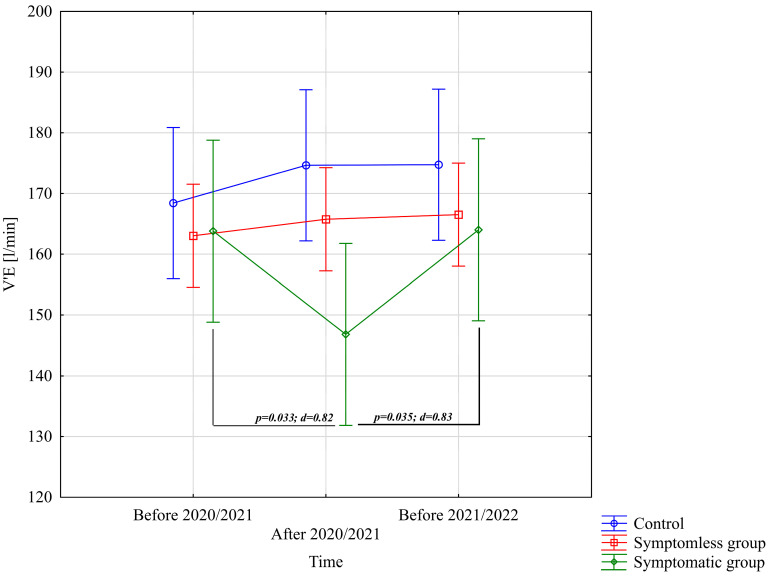
Comparison of mean values and confidence intervals for the V’E [L/min] variable by group and study time. *p*—*p*-value; d—Cohen’s d effect size.

The analysis of V’E [L/min] demonstrated significant differences only for the interaction between group and study time (*p* = 0.00073; η^2^ = 0.18). Further analysis of the group × study time interaction for the V’E [L/min] variable indicated significantly lower values in the WSG SpO_2_ < 90% group after the preparatory period 2020/2021 compared to the periods before the preparatory periods of both 2020/2021 and 2021/2022 (Figure 5).

**Figure 6:**
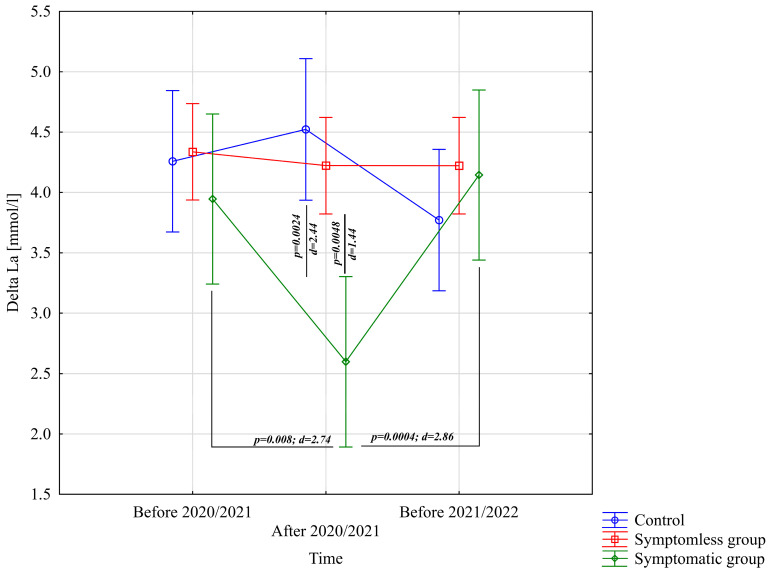
Comparison of mean values and confidence intervals for the ΔLa variable by group and study time. *p*—*p*-value; d—Cohen’s d effect size.

The analysis of ΔL [mmol/L] demonstrated significant differences for the main effect of study time (*p* = 0.011; η^2^ = 0.091) and the interaction between group and study time (*p* = 0.0002; η^2^ = 0.21). Further analysis of the group × study time interaction for the ΔL [mmol/L] variable indicated significantly lower values in the WSG SpO_2_ < 90% group after the preparatory period 2020/2021 compared to the periods before the preparatory periods of both 2020/2021 and 2021/2022 (Figure 6).

It was also observed that ΔL [mmol/L], VO_2Lt_ [mL/kg/min], VO_2_max [mL/kg/min], BF [1/min] and V’E [L/min] in the WSG group were recovered to baseline in next season.

## 4. Discussion

The most notable outcome of this study was the significant reduction in VO_2_max and VO_2_ at lactate threshold in athletes who experienced symptomatic COVID-19. Given that VO_2_max is critical for recovery between high-intensity intervals in ice hockey, this reduction may explain observed declines in overall performance. Importantly, it was also observed that, by the onset of the 2021/2022 season, athletes in the symptomatic group demonstrated a full return of selected physiological markers to their pre-infection baselines. Specifically, ΔLa, VO_2Lt_, VO_2_max, BF, and V’E all rebounded to values statistically indistinguishable from those recorded before any SARS-CoV-2 infection.

Although these findings were anticipated, concerns remained regarding the affected players’ ability to return to pre-illness performance levels or whether the disease changes would not leave irreversible changes that would prevent the players from continuing their careers. In both cases, in the group that suffered from COVID-19 symptomatically, unlike the groups that did not suffer from COVID-19 or asymptomatic COVID-19, the values of VO_2_max and VO_2Lt_ significantly decreased in a statistically significant manner, which caused very great concern due to the fact that the sport of ice hockey is characterized by multiple periods of high-intensity exercise interspersed with recovery periods. Shifts on the ice range from 45 to 60 s, so an aerobic system may be of prime importance for a recovery process [1,2,4,5,6,7,9,13]. According to Durocher et al., a high level of VO_2_max promotes rapid post-exercise recovery and prevents fatigue [24]. According to Green et al., a previous investigation with Division I college hockey players found that increasing VO_2_max improved the number of scoring chances and involvement on both ends of the ice [6]. Tescha and Wright [25] showed that the ability to recover from high-intensity exercise was highly correlated to capillary density and the resulting increase in oxygen supply to the fatigued muscles. Colliander [26] has shown that enhanced oxygen delivery to muscles post exercise potentially accelerates the rate of PCr resynthesis, an oxygen-dependent process. The work of Stanula et al. showed that aerobic capacity measured as VO_2_max was negatively correlated with special fitness tests on 6 × 89 m ice, both in terms of running times and fatigue index. Other researchers drew the same conclusions [25,26,27,28,29,30]. Stanula et al. stated that the relationships between VO_2_max and the fatigue index for repeated sprints found in this and the above studies seem to indicate that aerobic processes play a role in the recovery of energy substrates, which are necessary to exercise at high intensity. There are many mechanisms that can explain these results. Most of all, high aerobic power increases the ability to recover from repeated bouts of anaerobic power and probably decreases lactate concentrations in response to higher LA utilization in slow-twitch muscle fibers [25,31]. The analyses showed that the rate of lactate utilization after the VO_2_max test was also impaired in the symptomatic group, unlike the asymptomatic and control groups, in which the rate of lactate utilization after the preparatory period increased statistically significantly. According to the authors dealing with this topic [12,26,31], lactate removal from muscle is enhanced by increased buffering capacity and increased blood flow. Increased capillary density, as seen in endurance-trained individuals, provides a decreased diffusion distance between capillaries and muscle fibers, enhancing the movement of oxygen and nutrients and the removal of H+ and lactate from the muscle [32]. Enhanced oxygen delivery to muscles post exercise potentially accelerates the rate of PCr resynthesis, an oxygen-dependent process [26,33]. Players can optimize preseason VO_2_max values by performing off-ice training programs. In the case of people who became symptomatic with COVID-19, this goal was not achieved. To improve the oxidative capacity of muscle during the season, players may be able to employ sprint-interval training on a cycle ergometer (i.e., Wingate tests) as a way to effectively train while saving time. Sprint-interval training is also said to improve resting muscle glycogen content [34], which could be of great benefit to hockey players, who have been shown to deplete muscle glycogen by up to 70% during only 10 high-intensity simulated hockey shifts [35]. The results showed that only in the case of the control and asymptomatic groups, VO_2_max, VO_2Lt_, and the rate of recovery increased after the preparatory period, and thanks to the increasingly stronger league (the league open to foreigners), the levels of these determinants before the next preparatory period were at a high level. In the case of the symptomatic group, the level of VO_2_max, VO_2Lt_, BF, V’E, and ∆La were impaired after the preparation period. There was a concern that symptomatic players would not be able to return to their previous results due to the effects of the novel coronavirus. However, as the season progressed, the players demonstrated a restoration in their performance.

The decrease in VO_2_max, VO_2_ at the Lt threshold, and rate of recovery in the symptomatic group after COVID-19 infection is likely due to several mechanisms related to the pathophysiology of the disease. First, COVID-19 primarily affects the respiratory system, leading to inflammation and damage to the lungs. This can result in reduced lung capacity, impaired gas exchange, and decreased oxygen delivery to the body’s tissues, ultimately leading to decreased exercise capacity. Second, COVID-19 infection can also lead to widespread inflammation and damage to multiple organs, including the heart and vascular system. This can result in cardiac dysfunction, impaired blood flow, and decreased oxygen delivery to the muscles during exercise, leading to reduced exercise capacity. Third, COVID-19 infection can also lead to prolonged periods of inactivity and bed rest, which can result in muscle wasting, decreased cardiovascular fitness, and reduced exercise capacity. Finally, psychological factors such as anxiety and depression, which are common in patients with COVID-19, can also contribute to decreased exercise capacity and performance.

Overall, the decrease in VO_2_max, VO_2_ at the Lt threshold, and rate of recovery in the symptomatic group after COVID-19 infection is likely due to a combination of these factors, including lung and heart dysfunction, prolonged inactivity, and psychological factors. Further research is needed to fully understand the mechanisms underlying these changes and to develop effective interventions to improve exercise capacity and recovery in patients with COVID-19.

Based on the analyses, it was also found that in the control and asymptomatic groups, there was a significant increase in Relative Mean Power in the Wingate test between the results before and after the preparatory period, unlike the symptomatic group in which the level of Relative Mean Power statistically significantly decreased. The ice hockey players’ interval effort is based on 69% of anaerobic metabolism, during which the ice hockey player incurs an oxygen debt of 8.5–9.6 L, and there is a significant increase in lactate concentration to the level of 8–14 mmol/L [2,5,11]. It can be stated that Relative Mean Power indirectly describes the level of glycolytic capacity, which is a very important determinant of sports championship in ice hockey. Relative Mean Power is a determinant that has been taken into account in drafts for the best leagues in the world for many years [2,4,6,7,8,10]. When working on the ice, the effort lasts from 45–60 s, so from about 10 s, it activates mainly the glycolytic system, whose power peak occurs about 30 s after the effort. In this case, energy is generated from the breakdown of glucose from the blood and glycogen resources. Glucose utilization occurs without oxygen. This process is important for players playing at the highest level in ice hockey because shaping anaerobic capacity is not only about increasing the speed and power of a single movement, i.e., the so-called phosphagen power, but also the player’s ability to maintain maximum intensity while working on the ice, the highest possible phosphagen capacity, and glycolytic power. In the case of the symptomatic group, the level of Relative Mean Power decreased after the preparation period, and here, it was also feared that symptomatic players would not be able to return to their previous results due to COVID-19. But over the course of the league season, with the effort in match mode, the players rebuilt. The Relative Mean Power results before the next preparatory period did not differ statistically significantly in the analyzed groups.

Although speculative, potential explanations for reduced anaerobic performance in symptomatic athletes may involve COVID-19–related thromboembolic risk or subclinical myocardial inflammation, as suggested in previous studies [36]. However, our study did not assess biomarkers or imaging to support these mechanisms, and such interpretations should be viewed as hypotheses requiring further investigation. As a result of infection with the SARS-CoV-2 virus, blood clots can form, which can block blood vessels and impede the flow of oxygenated blood to tissues, including muscles. This can lead to tissue damage and impaired muscle function, potentially contributing to the reduction in “Relative Mean Power” scores seen in symptomatic athletes. It should be taken into account that the virus can cause damage to the lungs, causing deterioration of lung function and impaired oxygen uptake. This can lead to reduced oxygen delivery to muscle cells during exercise, contributing to decreased muscle strength and endurance. A decrease in SpO_2_, which measures the amount of oxygen in the blood, may additionally indicate impaired oxygen uptake and delivery, further reducing the “Relative Mean Power” scores. Treatments such as anticoagulant therapy and rehabilitation programs can help restore both thromboembolic mechanisms and oxygen limitation, leading to the restoration of “Relative Mean Power” in athletes. However, more research is needed to fully understand the mechanisms involved and develop effective treatments for athletes affected by COVID-19. In athletes who have recently had a viral infection such as COVID-19, myocarditis, or inflammation of the heart muscle, is a potential complication that can occur after strenuous exercise. SARS-CoV-2 can infect the heart and directly cause myocarditis, leading to inflammation and damage to the heart muscle. Symptoms of myocarditis can include chest pain, shortness of breath, fatigue, and palpitations. In severe cases, it can lead to heart failure and other complications. It is important for athletes, after recovering from a viral infection, to return to exercise gradually and undergo cardiac evaluations before resuming strenuous exercise. The analyzed groups did not significantly differentiate the results of the variables from the Wingate test, i.e., time to maximum power and Relative Mean Power, respiratory rate BF [1/min], and minute ventilation V’E [L/min] from the VO_2_max test. The Wingate test is a measure of anaerobic capacity and may not be as sensitive to the effects of COVID-19 on aerobic capacity and lung function. It is possible that testing time and recovery time after COVID-19 infection may have played a role in the lack of significant differences. The study may have been carried out too soon after the infection, before the athletes had fully recovered, or too late, after they had already returned to their pre-infection fitness levels. It is also possible that the athletes’ training and conditioning programs during the recovery period may have contributed to their ability to maintain their performance levels. The lack of significant differences may result from athletes compensating for reduced ventilation with increased respiratory rate. Such a compensatory mechanism may have resulted in a non-significant trend towards more breaths and less ventilation in the symptomatic group. Further research is needed to fully understand the impact of COVID-19 on athletic performance and lung function, and potential factors that may contribute to the observed differences or lack of them.

### Study Limitations

This study has several limitations. The sample size, particularly in the symptomatic group (n = 9), was relatively small, which may limit statistical power and generalizability. Although players followed team training programs, individual differences in training adherence, recovery, or psychological stress were not objectively monitored. Additionally, we did not collect biochemical markers of inflammation, myocardial injury, or hormonal response, which would help in exploring physiological mechanisms. Finally, the reliance on SpO_2_ as the main indicator of symptom severity may not capture the full clinical complexity of COVID-19 in athletes.

## 5. Conclusions

The results of this study clearly indicate that symptomatic SARS-CoV-2 infection exerts a significant negative impact on both aerobic and anaerobic performance parameters in elite ice hockey players. Athletes who experienced symptoms accompanied by oxygen desaturation (SpO_2_ < 90%) demonstrated marked reductions in VO_2_max and VO_2_ at lactate threshold (VO_2Lt_), suggesting compromised aerobic efficiency and limited capacity for physiological recovery between high-intensity efforts. These findings are particularly important in the context of a sport like ice hockey, where recovery between intense intermittent shifts is essential to sustained performance. In addition to the aerobic deficits, symptomatic players also showed significant impairments in anaerobic power output and lactate clearance. Relative Mean Power in the Wingate test was substantially lower in this group compared to both asymptomatic and control athletes, and post-exercise blood lactate concentrations remained elevated longer. This pattern points to reduced glycolytic efficiency and slower metabolic recovery, both of which can hinder the ability to perform repeated explosive efforts during a match.

In contrast, players in the asymptomatic group and the control group improved most physiological markers during the monitored period, reflecting the expected benefits of structured preseason training. Notably, by the onset of the 2021/2022 preparatory season, all key physiological metrics in the symptomatic cohort—ΔLa (lactate clearance rate), VO_2Lt_, VO_2_max, respiratory frequency, and minute ventilation—had returned to values statistically indistinguishable from pre-infection baselines. This full restitution underscores the considerable physiological resilience of elite athletes and suggests that, with appropriately graduated training protocols and individualized return-to-play planning, complete recovery from even hypoxemic COVID-19 is achievable. Based on the observed declines in both oxygen uptake and lactate utilization, sprint-interval training may be particularly beneficial as a rehabilitation strategy due to its ability to simultaneously stimulate aerobic and anaerobic adaptations. Future studies should aim to explore long-term physiological outcomes in larger athlete populations and assess additional factors such as inflammatory markers, cardiac function, and psychological well-being to better understand the full impact of COVID-19 on athletic performance.

## Figures and Tables

**Table 1:** Characteristic of the research sample.

Study Time	Variable	Group		
		NSG (n = 28)	CG (n = 13)	WSG (n = 9) SpO_2_ < 90%
		M ± SD	M ± SD	M ± SD
Before the preparation period 2020/2021	Height	184 ± 6.17	184.49 ± 5.20	181.96 ± 5.69
	Weight	86.08 ± 7.41	87.63 ± 6.01	85.47 ± 6.60
	% PBF	15.49 ± 4.72	15.48 ± 3.28	15.81 ± 4.18
After the preparatory period 2020/2021	Height	184 ± 6.17	184.49 ± 5.20	181.96 ± 5.69
	Weight	85.04 ± 7.38	86.99 ± 6.03	85.11 ± 6.58
	% PBF	14.52 ± 4.61	14.62 ± 1.48	15.31 ± 1.43
Before the preparatory period 2021/2022	Height	184 ± 6.17	184.49 ± 5.20	181.96 ± 5.69
	Weight	85.37 ± 7.23	87.64 ± 5.98	87.07 ± 6.92
	% PBF	14.63 ± 4.58	14.78 ± 1.48	15.02 ± 2.47

M—mean, SD—standard deviation. NSG—no symptoms group; CG—control group; WSG—with symptoms group.

**Table 2:** Comparison of means and standard deviations between the groups for the analyzed variables in 3 research periods.

Variable	Study Time	Group			Main Effects and Interaction for Anova with Repeated Measures F/*p*/η^2^
		NSG (n = 28)	CG (n = 13)	WSG (n = 9) SpO_2_ < 90%	
		M ± SD (95%CI)			
Time to Peak Power [s]	BPP 2020/2021	2.35 ± 0.96 (1.98; 2.73)	2.06 ± 0.62 (1.69; 2.43)	2.04 ± 0.81 (1.43; 2.66)	Group: F = 1.03; *p* = 0.36; η*_p_*^2^ = 0.042 Study time: F = 0.46; *p* = 0.63; η*_p_*^2^ = 0.009 Group × study time: F = 0.060; *p* = 0.99; η^2^ = 0.003
	APP 2020/2021	2.31 ± 0.89 (1.94; 2.69)	2.02 ± 0.62 (1.65; 2.39)	2.03 ± 0.81 (1.41; 2.65)	
	BPP 2021/2022	2.41 ± 0.91 (2.04; 2.79)	2.12 ± 0.62 (1.75; 2.49)	2.25 ± 0.81 (1.64; 2.87)	
Relative Mean Power [W/kg]	BPP 2020/2021	10.04 ± 0.86 (9.70; 10.37)	9.98 ± 0.38 (9.75; 10.21)	10.21 ± 0.37 (9.93; 10.50)	Group: F = 1.43; *p* = 0.25; η*_p_*^2^ = 0.057 Study time: F = 35.76; *p* < 0.0001; η*_p_*^2^ = 0.43 Group × study time: F = 6.26; *p* = 0.00016; η*_p_*^2^ = 0.21
	APP 2020/2021	11.21 ± 0.82 (10.88; 11.55)	11.12 ± 0.36 (10.90; 11.34)	10.00 ± 0.40 (9.69; 10.31)	
	BPP 2021/2022	11.31 ± 0.84 (10.98; 11.65)	11.22 ± 0.36 (11.00; 11.44)	10.97 ± 0.52 (10.57; 11.37)	
Relative Peak Power [W/kg]	BPP 2020/2021	17.84 ± 1.73 (17.17; 18.51)	19.22 ± 1.39 (18.38; 20.05)	19.15 ± 1.73 (17.83; 20.48)	Group: F = 3.26; *p* = 0.047; η*_p_*^2^ = 0.12 Study time: F = 11.67; *p* < 0.0001; η*_p_*^2^ = 0.20 Group × study time: F = 1.02; *p* = 0.40; η*_p_*^2^ = 0.042
	APP 2020/2021	19.04 ± 1.62 (18.37; 19.71)	20.46 ± 1.39 (19.62; 21.30)	20.30 ± 1.71 (18.99; 21.62)	
	BPP 2021/2022	19.14 ± 1.84 (18.47; 19.81)	20.56 ± 1.39 (19.72; 21.40)	19.36 ± 1.73 (18.04; 20.69)	
VO_2_max [mL/kg/min]	BPP 2020/2021	50.96 ± 3.87 (49.46; 52.47)	52.81 ± 5.35 (49.58; 56.05)	54.73 ± 5.09 (50.82; 58.64)	Group: F = 0.52; *p* = 0.58; η*_p_*^2^ = 0.021 Study time: F = 7.21; *p* = 0.0012; η*_p_*^2^ = 0.13 Group × study time: F = 17.96; *p* < 0.0001; η*_p_*^2^ = 0.43
	APP 2020/2021	54.56 ± 3.92 (53.04; 56.08)	56.17 ± 6.15 (52.46; 59.89)	48.22 ± 2.88 (46.00; 50.43)	
	BPP 2021/2022	53.50 ± 3.93 (51.97; 55.02)	56.27 ± 6.15 (52.56; 59.99)	54.94 ± 5.09 (51.03; 58.85)	
VO_2Lt_ [mL/kg/min]	BPP 2020/2021	39.52 ± 3.88 (38.02; 41.03)	41.57 ± 4.63 (38.77; 44.36)	43.10 ± 4.84 (39.38; 46.81)	Group: F = 2.39; *p* = 0.10; η*_p_*^2^ = 0.092 Study time: F = 40,13; *p* < 0.0001; η*_p_*^2^ = 0.46 Group × study time: F = 52.78; *p* < 0.0001; η*_p_*^2^ = 0.69
	APP 2020/2021	45.52 ± 3.81 (44.04; 46.99)	48.34 ± 4.84 (45.41; 51.26)	35.67 ± 4.47 (32.24; 39.10)	
	BPP 2021/2022	44.67 ± 3.43 (43.34; 46.00)	48.44 ± 4.84 (45.51; 51.36)	43.31 ± 4.84 (39.59; 47.02)	
BF [1/min]	BPP 2020/2021	53.19 ± 8.97 (49.71; 56.67)	50.10 ± 6.81 (45.98; 54.21)	49.07 ± 4.79 (45.39; 52.76)	Group: F = 0.66; *p* = 0.52; η*_p_*^2^ = 0.027 Study time: F = 2.65; *p* = 0.076; η*_p_*^2^ = 0.0053 Group × study time: F = 3.49; *p* = 0.011; η*_p_*^2^ = 0.13
	APP 2020/2021	50.91 ± 8.87 (47.47; 54.35)	48.10 ± 6.81 (43.98; 52.21)	58.52 ± 5.88 (54.00; 63.04)	
	BPP 2021/2022	50.90 ± 8.94 (47.43; 54.37)	48.20 ± 6.81 (44.08; 52.31)	49.28 ± 4.79 (45.60; 52.97)	
V’E [L/min]	BPP 2020/2021	163.03 ± 25.23 (153.25; 172.82)	168.41 ± 17.53 (157.82; 179.00)	163.81 ± 21.86 (147.00; 180.61)	Group: F = 0.59; *p* = 0.55; η*_p_*^2^ = 0.025 Study time: F = 2.83; *p* = 0.064; η*_p_*^2^ = 0.057 Group × study time: F = 5.28; *p* = 0.00073; η*_p_*^2^ = 0.18
	APP 2020/2021	165.74 ± 24.84 (156.11; 175.38)	174.64 ± 17.33 (164.17; 185.12)	146.81 ± 19.70 (131.66; 161.95)	
	BPP 2021/2022	166.52 ± 25.29 (156.71; 176.33)	174.74 ± 17.33 (164.27; 185.21)	164.02 ± 21.86 (147.21; 180.82)	
ΔL [mmol/L]	BPP 2020/2021	4.34 ± 1.18 (3.88; 4.79)	4.26 ± 0.99 (3.66; 4.86)	3.95 ± 0.54 (3.53; 4.36)	Group: F = 2.67; *p* = 0.079; η*_p_*^2^ = 0.010 Study time: F = 4.72; *p* = 0.011; η*_p_*^2^ = 0.091 Group × study time: F = 6.15; *p* = 0.0002; η*_p_*^2^ = 0.21
	APP 2020/2021	4.22 ± 1.26 (3.73; 4.71)	4.52 ± 0.95 (3.95; 5.10)	2.60 ± 0.44 (2.26; 2.93)	
	BPP 2021/2022	4.22 ± 1.12 (3.79; 4.66)	3.77 ± 1.19 (3.05; 4.49)	4.14 ± 0.62 (3.67; 4.62)	

M—mean; SD—standard deviation; 95%CI—95% confidence interval. F—Fisher’s F-statistic; *p*—*p*-value; η*_p_*^2^—partial eta-squared; BPP 2020/2021—Before the preparation period 2020/2021; APP 2020/2021—After the preparatory period 2020/2021; BPP 2021/2022—Before the preparatory period 2021/2022. VO_2_max—maximal oxygen uptake; VO_2Lt_—oxygen uptake at lactate threshold; BF breathing frequency; V’E—minute ventilation; ΔL—change in blood lactate concentration; NSG—no symptoms group; CG—control group; WSG—with symptoms group.

## Data Availability

The datasets used and analyzed during the current study are available from the corresponding author upon reasonable request.
